# Obesity and environments external to the body

**DOI:** 10.1098/rstb.2022.0226

**Published:** 2023-09-11

**Authors:** Stanley Ulijaszek

**Affiliations:** Unit for BioCultural Variation and Obesity, School of Anthropology, University of Oxford, Oxford OX2 6PE, UK

**Keywords:** food, built environment, sociality, polycentric, body fatness, obesity

## Abstract

Studies of environment and obesity usually use epidemiologically tractable measures that are proxies for energy balance or macronutrient composition intake, mostly to identify individual behavioural changes for prevention or reduction of obesity, or inform policy. Of environments external to the body as they relate to obesity, the built environment and the food environment are considered among the most important. Incorporating human sociality into obesity and environments research enriches the field by offering possible ways for understanding obesity production via social stress, dietary preference, food consumption and physical activity. External environments are in flux, however, especially with changing urban form and social environmental hybridity since Web 2.0, with urban polycentricity and networked and online activity influencing obesity production in new ways. While the world's rural populations are experiencing the fastest increases in obesity, large urban populations benefit from scale in setting the physical conditions for physical activity and healthy food availability, with larger and polycentric cities having lower rates of obesity than smaller monocentric or dispersed cities. It is argued that built, food and social environments set the context for obesity production or its amelioration, with sociodemographic factors being more important than new phenomena such as digital and smart technologies.

This article is part of a discussion meeting issue ‘Causes of obesity: theories, conjectures and evidence (Part I)’.

## 1. Introduction

Biological susceptibilities to obesity can only be expressed in environments where it is easy for energy intake to exceed energy expenditure, and/or where the macronutrient composition of the diet is conducive to weight gain. Towards the aim of finding environmental factors in obesity production, epidemiologically tractable measures which act as proxies for energy balance are usually used for either identifying individual-level behavioural changes for intervention [1], or to inform policy [2]. In public health, obesity has been framed as an epidemic, which forms, alongside epidemics of undernutrition and climate change, a so-called Global Syndemic affecting most people across the world now, co-occurring in time and place with complex outcomes and sharing common underlying societal drivers [3].

Understanding environmental factors in obesity production is also important for understanding and framing this Global Syndemic. A focus on behavioural change accepts individualist norms of western society, while a focus on policy accepts society as the appropriate level of intervention against obesity. A dominant framing of environment in obesity studies now is a twofold one, of food and of physical activity [4], set within broader contexts of external factors known to be related to obesity [5,6]. The vast majority of the world's population now lives in built environments [7], and these influence patterns of physical activity and of food access [8], as well as sociality. Obesity emerges and is propagated in social contexts [9,10], mediating body norms, physical activity and food intake [11]. The dual materialities of the built environment and of food are considered here in relation to social processes that influence the production of obesity.

Techno-social change across the past two decades with the emergence of Web 2.0 has seen digital environments transform patterns of human sociality beyond the physical constraints of local built environments. This has continued to change how both built and food environments are negotiated [12]. This review thus also considers how the digital environment influences individual behaviour in the production of obesity. The article begins by considering how the environment is framed by researchers in relation to obesity, going on to consider how built, food and social environments are enmeshed in the production of obesity.

## 2. Setting the boundaries

**Figure 1.**
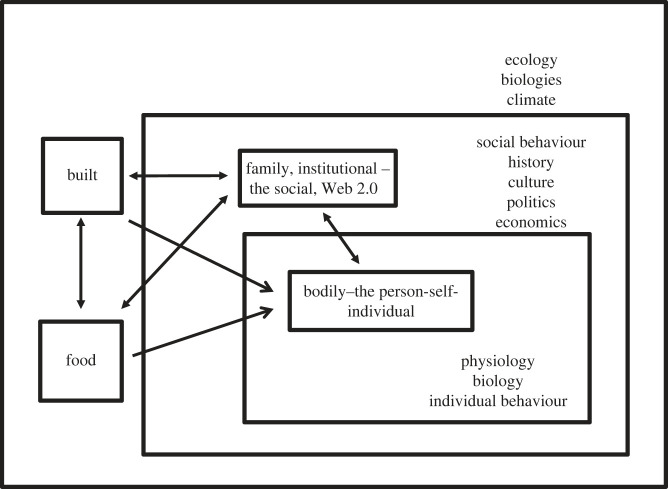
Environments relating to obesity.

Boundary-setting in obesity-environment studies separates the object of study, whether individual, community or society, from the world around it. There are many ways of doing this, including Swinburn *et al.*'s [5] framing of obesogenic environments and Drewnowski *et al.*'s [4] modelling of obesity in the built environment. Machine reading and semantic analysis of scientific literature on obesity and environment has helped identify five distinct ways in which researchers frame the environment in obesity research (table 1). These are bodily, familial, food, built and institutional [6], each carrying particular assumptions, traditions of research, and conceptualizations of what is legitimate to study within the field overall. They are located at different scales, from the internal bodily environment, to familial, community, regional, national and global environments (figure 1).

**Table 1.** Research approaches to environment and obesity (adapted from Jensen *et al*. [6] and Ulijaszek [11]).

environment	focus	mechanism	societal framing
bodily	fat deposition in human or rodent organisms	physiological processes, gene environment interactions, epigenetics	science
familial	population obesity in children and adolescents	energy expenditure and intake	people
institutional	institutional food services	policies and their implementation	government
food	population obesity	energy intake	corporations
built	population obesity	energy expenditure	urban planning

Bodily environments include the intracellular milieu for the metabolic and endocrine regulation processes that can predispose to weight gain, as well as metabolic dispositions endowed in fetal and early development. Internal bodily environments interact with bodily external ones, including social, domestic, societal and built spheres of daily individual engagement. The gut environment is situated between internal environments that require nutritional support to be able to function, and a microbiome which is part of a body ecology comprising of the biological relationships among the gut, its contents, and the brain [13]. Extra-bodily environments are most immediately experienced domestically (the family environment), and then in the wider world (food, built and institutional environments). Both domestically and more broadly, individuals engage in social environments which influence how body size and shape are experienced, and how the physical environment and food environment are negotiated [14]. More broadly yet, environments are structured politically, economically and physically at different social and political levels, influencing the production of obesity through interactive relations. For this article, the boundary is set around the individual body and environments external to it. This static materialist representation is made dynamic by engaging the social environment as a mediator of obesity production [15]. Observing obesity as a social phenomenon brings the possibility of it being framed as a complex socially networked phenomenon [9] that transcends place, along with factors known to be associated with it, including physical activity [16], stress [17] and food choice [18].

## 3. External environments: built, food and social

In obesity and environment research, the built environment is distinguished from the natural environment by its human-made materiality—its vehicular and pedestrian infrastructure, its buildings, its public places—as well as being the locale of domestic life, work and leisure [7]. While not as strongly related to obesity as genetic, epigenetic and behavioural factors [7], the built environment is nonetheless a strong external influence. In 1945, politician Winston Churchill observed that ‘We shape our buildings and afterwards our buildings shape us’ [19, p. 1]. On a larger scale, Danish architect and urban designer Gehl [20, p. 9] has stated that ‘First we shape the cities—then they shape us.’ Patterns of physical activity, eating and sociality are shaped through ways in which urban space is configured and organized; types and organization of built and natural features within cities; and forms of transportation and logistics [21]. Built environments range in scale from individual buildings to neighbourhoods, towns and cities, and include supporting infrastructures such as water supply, energy networks [22], transportation, and the internet. They have been defined as material, spatial and cultural products of human labour that combine physical elements and energy in forms for living, working and playing [23]. Built environments are material and cultural artefacts, outcomes of human practice and behaviours past and present, which in turn act upon and shape human ecology and biology, including the shaping of bodies and the production of obesity.

In epidemiological studies of obesity, food environments are usually reduced to the presence of food relative to individuals and populations through physical proximity to food store locations, the distribution of food stores, food services, and any physical entity by which food may be obtained, or any connected system that allows access to food [24]. This privileges physical exposures to food above forces like marketing and branding, which often mediate purchase and consumption externally to locales of food presence [25]. Sensory cues that can stimulate people to over-eat in the built environment [26], including ubiquitous exposure to food and the omnipresence of food advertising [27], are also important, but usually not considered by epidemiologists.

### (a) Urban form, scale and obesity

The early observation that at country level, average body mass index (BMI) has increased in tandem with urban growth informed the view that urbanization is one of the most important factors in the global rise of obesity [28]. This view has since been disrupted by evidence that the majority of the global rise in mean BMI between 1985 and 2017 has taken place in rural areas, overwhelmingly so in low- and middle-income countries [29].

By the turn of the twenty-first century, cities, towns, rural hinterlands and untamed wilderness areas of the world had become integrally bound in urban form by virtue of proximity and linkages [30], with the majority of the world's population living in built environments soon after [7]. Among factors that influence differences in obesity rates, socio-demographic factors are well-studied, and clearly discriminate obesity rates by socio-economic status (SES) and urban deprivation [31]. In the United States (USA), obesity levels are higher in rural areas (defined as counties with a metropolitan centre of population less than 50 000) than in urban areas [32]), being associated more strongly with sociodemographic factors than negative aspects of urbanism. Since 2000, poverty has increased most in rural counties of the USA [33], while rural–urban differences in obesity have been shown to be associated mostly to differences in educational level at the individual level, and economic and built environmental differences at the neighbourhood level [34].

Urban size and shape are important too. Within cities of the USA, compact areas have lower rates of obesity than sprawling ones, mostly due to differences in walkability and other possibilities for engaging in physical activity [35]. The bigger a city is the more complex it can be. Income and *per capita* gross domestic product (GDP) have been shown to be proportionately higher relative to population size, while the scale of infrastructure—road surface, petrol stations, electricity cables—proportionately lower [36]. Resources that relate directly to immediate human need, things like housing, employment, electricity and water supply, scale in linear fashion [36]. Scaling exponents of urban size also vary according to GDP, innovation, consumption and intensity of public transport, with complementary factors simultaneously present allowing the existence of phenomena in larger cities which are less prominent in smaller ones [37]. For example, levels of educational attainment and extent of employment in industry both scale in super-linear fashion with population size, being complementary factors to employment levels in innovation. Super-linear scaling also holds true for infectious diseases, with spread being faster in larger cities for HIV/AIDS in the past [36] and COVID-19 in the early stages of the pandemic [38].

To examine whether obesity rates scale inversely with urban size, an analysis is carried out here for 181 urban centres in the USA. Population data from the Census of 2010 [39] is related to metropolitan obesity rates obtained in the Gallup Healthways Survey of 2011 [40] for populations of metropolitan statistical areas considered to be urban (greater than 50 000 people). In this urban population sample, obesity rates are lower in larger metropolitan areas than smaller ones (*t* = 3.33, *p* < 0.001), and negatively related with population size (*F* = 10.36; *p* = 0.0015; *r* = −0.23). Obesity also scales linearly (*β* = 0.99) with urban size rather than exponentially or logarithmically in a similar way to factors associated with human need and function, like housing, employment, electricity and water supply.

Most of the larger conurbations in the USA are now polycentric, following a global trend in urban growth across the past two decades or so. Polycentric urbanism involves multiple independent centres with similar degrees of importance [41]. In the USA, Yang & Zhou [42] have shown that polycentric built environments have lower obesity rates, attributing this difference to differences in physical activity. In China, polycentric urban structures facilitate physical activity to a greater extent than do neighbourhood-level factors [43]. Polycentric structures offer greater spatial variability of neighbourhood-level density, and of infrastructure such as street connectivity and land use mix [43], both of which promote physical activity and access to healthy food through efficient supply infrastructures. In addition to differences in physical activity, lower obesity rates in polycentric cities may be associated with greater population densities, higher *per capita* incomes and lower poverty rates relative to monocentric and dispersed cities [44]. Urban population concentration through polycentricity involves lower dependence on the motor car, offers more healthy eating through economies of scale in the distribution of healthy foods [45] and higher local turnover of such foods, which often have short shelf-lives. Urban polycentricity can thus mitigate against obesity, especially through active transport and physical activity at neighbourhood and inter-neighbourhood levels [43].

### (b) The neighbourhood

A common form of urban organization in obesity environment studies is that of the neighbourhood, largely because of its importance as a spatial unit in intervention in public health more broadly [46]. Neighbourhoods are spatial units in which daily face-to-face interactions are common, and where social control in general is high [47]. In obesity studies, neighbourhoods are largely partitioned into the food environment and the physical activity environment [4]. The food environment is conceptualized in terms of physical access to food resources—especially supermarkets, grocery stores, fast food restaurants and convenience stores. Physical activity environments are mostly conceptualized in terms of area walkability, access to green and blue spaces, availability of recreational facilities, and land use mix. These conceptualizations are then reduced by epidemiologists to ‘exposures of interest’ in relation to where people live, which can then be quantified as forms of poor diet and/or lack of exercise, and statistically analysed in relation to body weight, BMI, or obesity rates. The overwhelming focus on quantifying the built environment as exposure, together with cross-sectional study designs [4], has limited the possibility of drawing causal inferences from potential links between ‘exposures of interest’ and obesity [48], especially because much data collection is short-term, while obesity is a long-term outcome.

The focus on neighbourhoods for the production of policy-appropriate research is understandable, but misses many dynamics of human activity that may influence the production of obesity. For example, much food buying takes place outside of a neighbourhood of residence [49]; one study in the UK has shown that work and commuting environments contribute more to the food environment of working adults than do their neighbourhoods [50]. Furthermore, people using public transport to get to work are more physically active than those who are not, regardless of neighbourhood walkability [51]. Potential interventions at neighbourhood level are viewed as being important, however, because if successful they are deemed more likely to stick because of the high degree of social control within them [47]. Social control, is, however, associated with obesity stigma [52], while obesity stigma undermines neighbourhood social engagement among those with excess weight, especially women [53].

Studies of neighbourhood built environment show walking, green space, active transport and recreational facilities to be most closely negatively related to obesity, relationships between local food environment and obesity less so [4,54]. SES is more strongly negatively associated with obesity than physical proximity to food sources, whether these be supermarkets or fast-food restaurants [54]. In the USA, human behavioural and urban design features most closely inversely related to obesity include quality of walkability, having neighbours that walk to work, and living in a neighbourhood with older homes, built before 1940 [8]. A cultural bias in food environments research has been the now-challenged assumption that supermarkets always provide healthy food options [55]. A reframing of food environments that removes this bias sees them as involving interrelated external and personal domains [56]. The former refers to factors that influence food acquisition and consumption that are not directly influenced or controlled by individuals: food availability in stores; food prices; characteristics of food products and food vendors available; and marketing and regulation. The latter includes factors more directly related to individual agency: physical access to food; affordability; convenience; and individual preference, taste and knowledge.

Within neighbourhoods, domestic space is largely left undisturbed by researchers of obesity and environment, except in relation to child-rearing [57] and television use [58]. A study of physical activity in the domestic space among young children in the USA has revealed how this is related to family routine and activities of care, and to availability (or not) of domestic outside space, including yards and private gardens [59]. The various lockdowns of the COVID-19 pandemic pushed public health firmly into the domestic arena, a domain of food and built environment research more usually linked to social psychology.

### (c) Sociality

Humans perform their lives in social and societal environments [60], and social stress is an environmental factor that can contribute to obesity via dietary preference and food consumption [61]. Psychosocial stress due to low social position [62,63], greater social mobility [64] and weight stigma [65] is associated with less healthy dietary behaviour and with greater body weight, to a greater extent in women than in men [63]. Social and evaluative stressors most strongly engage the hypothalamus-pituitary-adrenal axis [66]. This is a central mechanism in obesity production, operating through appetite, food preference, sleep duration and physical activity patterns [63,65], both within- and across generations [67]. Psychosocial stress is associated with having to negotiate large social groups [68], and with extensive daily digital media use [69]. More directly, social media influence the production of obesity in the USA through social interactions over food posting, more so than with easy access to fast food restaurants, social approval favouring unhealthy food high in sugar [70].

Online activity has been shown to shape eating behaviours [71], bodily dissatisfaction [72] and physical activity patterns [73]. Obesity travels through social networks [9], and social media promote its travel. Three inter-related processes are viewed to drive this process: social contagion (whereby the network in which a person is embedded influences their weight or weight-influencing behaviours); social capital (whereby a sense of belonging and of having social support influences weight or weight influencing behaviours); and social selection (whereby a person's network might develop according to their weight) [74]. The digital environment is social, agentive, and influences behaviour. Use of screen media has been shown to influence obesity production in children and adolescents through: increased eating while viewing; exposure to high-calorie, low-nutrient food and beverage marketing which can influence food preferences, purchase requests and consumption habits; and reduced sleep duration [75]. New digital media are evolving fast, television now being almost a thing of the past, and new platforms and digital devices are proliferating. Diverse mobile technologies (since the launch of Web 2.0 in 2004) and the Internet of Things (since 2014) are disrupting and changing how built and food environments are negotiated, and it is unclear how pertinent earlier findings relating to passive screen-time and obesity are to understanding how obesity relates to interactive screen-use with the accelerated proliferation of digital technologies since 2004.

## 4. Conclusion

Many aspects of the external environment are related to obesity [76,77], the built environment and the food environment being considered among the most important [4]. The materiality of built and food environments is given meaning through human sociality, and social stress can contribute to obesity via metabolism, dietary preference, food consumption and physical activity. Social adversity and insecurity are enmeshed in the production of obesity at several environmental levels [67].

There is a strong focus on the neighbourhood level in obesity and environment research, largely because much public health policy and intervention takes place at this level. People do only part of their business at the neighbourhood level, however, and commuting to work, mobility through motor car use for shopping and recreation makes the neighbourhood a less-powerful shaper of behaviours that influence obesity now than it might have done in the recent past and especially since the rise of social media. Much of what is done in this type of research involves counting features that can be used in statistical analysis—food stores, pavements, parks—rather than how they are negotiated by people, reflecting an individualist bias. With respect to physical activity and environment, the ecological model of Bauman *et al*. [78] is most persuasive at the individual level, because of greater strength of evidence in individualist approaches in epidemiology and public health, but it reveals much less of what influences physical activity levels at the societal and global levels [21]. With respect to food, knowing what is sold and eaten where, understanding how food is chosen and how its materiality is turned into bodily metabolism, are both important, but without incorporating the dynamics of eating, little is revealed of how eating produces obesity. For example, some people eat in isolation, some eat socially, and both can be good or bad in measure. Moreover, most people eat at home most of the time, and while most of what is bought in a store is taken home and eaten there, there is less knowledge of who eats what within a home.

Large urban populations benefit from aspects of scale in setting the physical conditions for physical activity and healthy food availability. Polycentric cities have lower rates of obesity than monocentric and dispersed ones, and polycentric urban development is growing world wide [79]. In large polycentric urban places, smartness and use of smart devices should favour new forms of sociality and influence health-related behaviours [21]. While a polycentricity approach to obesity would regulate its production through macro-level urban planning, a neighbourhoods approach relies more on regulating social norms. Across the past two decades, life has become increasingly lived in online–offline hybridity, with mobile technology becoming incorporated into ways of life [80]. The platformization of society and everyday life [81] has implications for how environments external to the body can influence the production of obesity, in relation to shifting human cognition with changes in attentional capacity and social cognition [82], as well as in relation to negotiating the world through aesthetic and embodied capital [21]. While much is known of environments external to the body and obesity, these environments are in continual flux, with both changing urban form and increasing digital–physical hybridity of sociality influencing these relationships.

## Data Availability

Data used are secondary from existing publications, sources of which are referred to in the review. Available from the author upon request.
